# Unprecedented simultaneous enhancement in damage tolerance and fatigue resistance of zirconia/Ta composites

**DOI:** 10.1038/srep44922

**Published:** 2017-03-21

**Authors:** A. Smirnov, J. I. Beltrán, T. Rodriguez-Suarez, C. Pecharromán, M. C. Muñoz, J. S. Moya, J. F. Bartolomé

**Affiliations:** 1 Instituto de Ciencia de Materiales de Madrid (ICMM), Consejo Superior deInvestigaciones Científicas (CSIC), C/Sor Juana Inés de la Cruz 3, 28049 Madrid, Spain; 2 Moscow State University of Technology “STANKIN”, Vadkovskij per. 1, Moscow, 101472, Russian Federation; 3 Element Six UK Ltd, Global Innovation Centre, Fermi Avenue, Harwell Oxford, Didcot, OX11 0QR, UK; 4 Nanomaterials and Nanotechnology Research Center (CINN), CSIC-University of Oviedo (UO), Avda de la Vega 4-6, El Entrego, 33940 San-Martín del Rey Aurelio, Spain

## Abstract

Dense (>98 th%) and homogeneous ceramic/metal composites were obtained by spark plasma sintering (SPS) using ZrO_2_ and lamellar metallic powders of tantalum or niobium (20 vol.%) as starting materials. The present study has demonstrated the unique and unpredicted simultaneous enhancement in toughness and strength with very high flaw tolerance of zirconia/Ta composites. In addition to their excellent static mechanical properties, these composites also have exceptional resistance to fatigue loading. It has been shown that the major contributions to toughening are the resulting crack bridging and plastic deformation of the metallic particles, together with crack deflection and interfacial debonding, which is compatible with the coexistence in the composite of both, strong and weak ceramic/metal interfaces, in agreement with predictions of ab-initio calculations. Therefore, these materials are promising candidates for designing damage tolerance components for aerospace industry, cutting and drilling tools, biomedical implants, among many others.

3Y-TZP is an extensively used material for many structural applications due to its good mechanical performance, which is related to the tetragonal to monoclinic phase transformation of ZrO_2_ and it is associated to the volume expansion, 3–5%, and shear strain ≅7%^1^. This volumetric expansion generates stresses in the ceramic matrix, which hinders the crack propagation. Nonetheless, zirconia based ceramic materials are not suitable for applications under severe loading conditions due to insufficient surface finish or cracking induced during service and mishandling, being the main reasons for unpredictable failure of these ceramic components. Thereby, the presence of any type of bulk discontinuities or tiny defects may reduce their reliability.

Brittle ceramics matrix can be toughened by adding a ductile second phase to them. Cermets are ideally designed to combine the optimal properties of both, high wear resistant ceramics and ductile metals, which possess the ability to reduce crack propagation and prevent catastrophic failure.

However, these improvements are often achieved at the expense of strength. In these structural materials strength and toughness are usually considered mutually exclusive^2^. In terms of microstructural design, requirements for high strength are often different or even contradictory to those for high fracture toughness.

Several studies on zirconia reinforced with metals such as nickel^3^,^4^,^5^,^6^, stainless steel^7^,^8^,^9^, molybdenum^10^, titanium^11^, tungsten^10^,^12^, chromium^13^, iron^13^ have been reported in the literature. The mechanical properties of these ZrO_2_-based cermets reported by diverse scientific studies are substantially scattered. Exemplarily, the flexural strength (σ_*f*_) and fracture toughness (*K*_*Ic*_) of zirconia/metal composites described in the literature reach values up to 1200 MPa and 5.9 MPa∙m^1/2^ for ZrO_2_/Ni cermet^4^ and 310 MPa and 5.1 MPa∙m^1/2^ for ZrO_2_/Ti cermet^11^, respectively. That could be explained by the variations in initial compositions, distribution of reinforcement phase, processing and sintering conditions. Furthermore, the thermal expansion and elastic modulus of the different phases^14^,^15^, the particle-matrix interfaces^16^,^17^ and their structural anisotropy are important parameters. Moreover, it has been shown in many previous studies that the mechanical response of transition metals depends primarily on its purity, particularly regarding the oxygen, nitrogen, hydrogen and carbon content. The purity of the metal powder, the manufacturing process and the level of deformation are other factors that influence the final material properties^18^,^19^,^20^,^21^,^22^.

This suggests that a more refined understanding of the interrelations between different strengthening and toughening mechanisms is needed to optimize ceramic/metal composite microstructures for structural applications.

Additionally, embedding a ductile metallic phase into a brittle matrix increases not only their fracture toughness and damage tolerance, but also influences the fatigue performance^23^,^24^. However, to date, studies on the mechanical properties of such ductile-particle reinforced brittle materials have mainly focused on strength and fracture toughness behaviour; in particular on the contributions to toughening under monotonic loading conditions^25^,^26^,^27^,^28^,^29^. Conversely, very few investigations have been focused on the composite behaviour under cyclic load. These studies have shown that toughening is far less effective in fatigue simply because the ductile phase fails prematurely. Indeed, the fatigue-crack growth properties are often similar, and sometimes worse, than those corresponding to the unreinforced matrix^30^,^31^,^32^. Properties such as toughness (damage tolerance) and fatigue resistance are generally mutually exclusive. Therefore, fatigue analysis and fatigue strength prediction are highly required especially in the case of fail safe or damage tolerance design for components in spacecrafts and rocket engines, cutting and drilling tools, fuselage of supersonic planes, biomedical implants, among many others. Accurate prediction of fatigue life is a challenge in ceramic/metal composites due to the complicated nature of fatigue crack initiation and propagation, interfaces and complex material behaviour under loading and unloading regimes.

Recently, we have demonstrated that wet mixing route and hot press sintering tailored suits the fabrication of zirconia/Nb^16^ and zirconia/Ta^23^ composites with a flexural strength of 800 MPa and 992 MPa, and fracture toughness of 15 MPa∙m^1/2^ and 16 MPa∙m^1/2^, respectively. In the present work, we focus on the interplay between the damage tolerance and fatigue resistance phenomena within zirconia/Nb and zirconia/Ta composites.

## Results

### Microstructure

**Figure 1:**
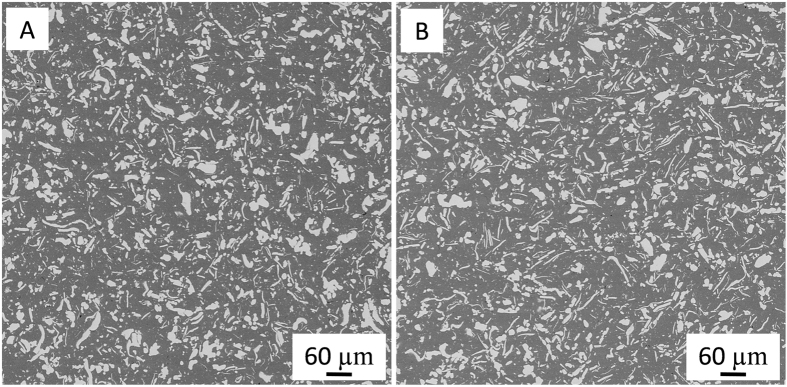
Scanning electron images corresponding to the microstructure of (**A**) zirconia/Ta and (**B**) zirconia/Nb composites. Darker and lighter phases are zirconia and the corresponding metal, respectively.

Scanning electron micrographs corresponding to ZrO_2_/metal composites are shown in Fig. 1. In these micrographs, the darker and bright phases correspond to zirconia and metal grains, respectively. The metallic particles are uniformly dispersed in the matrix and no porosity is observed. In the present work, the larger metallic particles are preferentially oriented due to the effect of the applied pressure during spark plasma sintering process. These flake shaped metal particles will be textured normal to the crack propagation. As can be seen, ZrO_2_/Ta and ZrO_2_/Nb interfaces are well bonded and no microcracks are observed. It has only been observed that a solid solution of Nb_2_O_5_ and Ta_2_O_5_ in ZrO_2_ takes place^33^,^34^. This solid solution of niobia and tantala in the zirconia matrix were calculated from the EDX spectra. These fractions were estimated to be 0.9 ± 0.4 and 1.4 ± 0.4 mol. %, respectively. Therefore, we assume that the entire passivation layer of Ta_2_O_5_ and Nb_2_O_5_ that is always present on the particle surfaces of the metal starting powder is dissolved in a solid solution in the zirconia matrix after sintering. On the other hand, it may well be that the oxygen is dissolved and distributed statistically in the metal^35^. However, no new suboxide has been detected.

**Figure 2:**
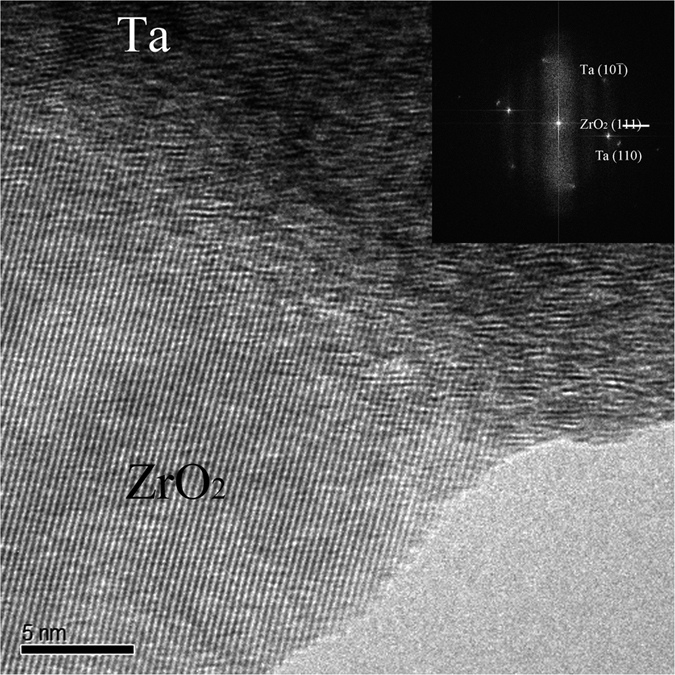
High resolution transmission electron micrograph of the ZrO_2_/Ta interface.

A representative high resolution transmission electron micrograph corresponding to ZrO_2_/Ta interface is shown in Fig. 2. This micrograph reveals a direct contact between both grains at the interfaces without any additional phases.

### Mechanical properties

The results related to the mechanical evaluation of the composites are enclosed in Table 1.

The mean biaxial flexural strength values corresponding to ZrO_2_, ZrO_2_/Ta and ZrO_2_/Nb were found to be 1217 ± 10 MPa, 970 ± 18 MPa and 850 ± 20 MPa, respectively. As a direct consequence of the smaller critical grain size in ZrO_2_, the bending strength of the monolithic ceramic is higher than the strength corresponding to both zirconia/metal composites. The Young’s moduli of the composites (≈200 GPa) were found to be very close to the values predicted by the rule of mixtures by Voigt and Reuss models. The average fracture toughness of ZrO_2_/Nb and ZrO_2_/Ta composites was found to be 15 ± 1 and 16 ± 0.9 MPa·m^1/2^, respectively; much higher than the value obtained for the monolithic zirconia (6 ± 0.3 MPa·m^1/2^).

**Figure 3:**
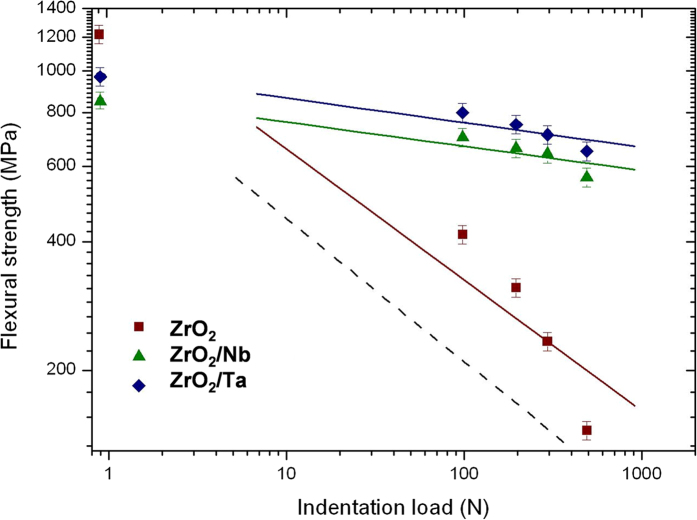
Indentation load versus strength plots of 3Y-TZP/Ta, 3Y-TZP/Nb composites and zirconia ceramic. The indentation-strength data to the P^−1/3^ strength response is shown by the diagonal dashed line.

In Fig. 3, a chart of the indentation load versus the strength of indented samples is plotted. Each data point represents the mean value of about twelve specimens tested at a given load. Linear fitting was applied and it was found that the slopes of monolithic zirconia and ceramic/metal composites were 0.30 and 0.06, respectively.

**Figure 4:**
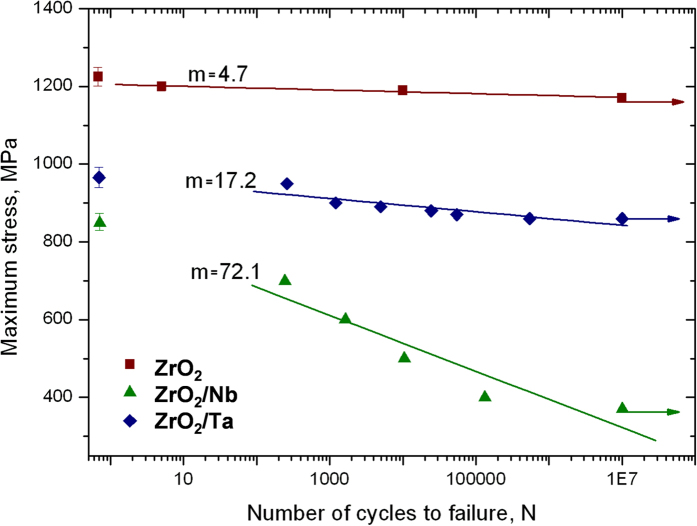
Fatigue resistance S–N curves with values of slopes for un-indented polished specimens.

Fatigue is undoubtedly a very important type of loading for many components containing dissimilar systems. In a fatigue loading regime, a structure may fail at a small percentage of its fracture strength. The results for cyclic fatigue life for the specimens tested with a disc geometry are presented in a semi logarithmic form in Fig. 4 as peak stress versus cycles to failure (*N*). The tests were interrupted at *N = *10^7^ cycles, for the unfailed samples, which are marked with an arrow symbol. Three maximum stress levels (*σ*_max_) were selected in relation to the initial strength obtained under static tests (Table 2). For the interpretation of materials fatigue data the exponential model was used. The estimation of the model parameters was based on linear regression analysis. It was found that the fatigue limit for ZrO_2_, ZrO_2_/Ta and ZrO_2_/Nb is 1200 ± 15 MPa, 860 ± 30 MPa and 370 ± 30 MPa, respectively (Table 2).

## Discussion

A detailed analysis of the micrograph in Fig. 2 allowed us to determine the presence of (111) crystallographic planes corresponding to the equivalent zirconia crystals parallel to the Ta (110). Additionally, Ta (10–1) planes were also detected (see the FT image inset in Fig. 2), so it can be concluded that <111> direction of Ta crystal is perpendicular to the picture plane. In this regard, a perpendicular plane to the {110} and {111} families is the plane Ta(0-1-2). Considering the geometry in Fig. 2, the only plausible ZrO_2_ planes parallel to this one are the ZrO_2_{110}. So that, we can assign the interface in the micrograph to be Ta(1-1-2)/ZrO_2_(3–30). This interface has a mismatch of 16% and has not been previously observed in the similar system ZrO_2_/Nb^16^. In this sense, the presence of (012) cleaving planes in milled Ta grains, could be the origin of such interface orientations^36^. In addition, due to the lamellar shape of Ta particles, it is expected that different ZrO_2_ crystallographic orientations form interfaces with the Ta (100) surface, as it occurs in ZrO_2_/Nb composites^16^. In fact, ab-initio calculations of the bonding strength and stability of several plausible ZrO_2_/Ta interfaces indicate that the experimentally observed (3–30)/(1-1-2) interface is thermodynamically stable and its bonding strength, measured by the work of separation (Wsep)^37^,^38^, is moderated (see Fig. S1 and Table S1 of Supplementary Information, SI). While the (100) ZrO_2_/(100) Ta polar interface presents the largest bonding strength, although its stability is lower. The Wsep of the representative investigated interfaces ranges from high (~10 J/m^2^) to medium values (~2 J/m^2^) (see SI for a discussion of the ab-initio calculations). Therefore, ZrO_2_/metal composites show diverse interfaces with different strength, being the strongest less stable and, consequently, the composites show a moderate average interface strength. As a result, the metal particle-matrix bond should be strong enough for load transfer and energy dissipation by metal particle plastic deformation but it should remain sufficiently weak to support cleavage and therefore act as a crack entrapment if the metal particle is large or with low plasticity.

The increase in toughness must be due to the ductile metallic phase, which can absorb the crack propagation energy during fracture, and could enhance crack deflection and bridging as well as stress relaxation near the crack tip. Moreover, this increase has occurred due to the presence of solid solution of pentavalent oxides such as Nb_2_O_5_ and Ta_2_O_5_, which enhance the transformability of zirconia^39^,^40^. In the present work, as a consequence of high oxygen avidity of zirconia, Nb and Ta flakes might be free of oxygen in solid solution. On the other hand, the fracture surface roughness of both composites (ZrO_2_/Nb and ZrO_2_/Ta) sintered in a C free atmosphere (Ar atmosphere)^29^,^41^, as well as, in this study, under SPS condition (using graphite dies) were found to be almost identical. Therefore the presence of O_2_ and C impurities doesn’t affect significantly the ductility of Ta and Nb flakes.

Additionally, the effects of the residual stresses related to the CTE mismatch between zirconia and the metals case of study have to be taken into consideration. The average thermal expansion coefficient, in the 20–1000 °C range, is 6.73 × 10^−6^ °C^−1^ and 7.3 × 10^−6^ °C^−1^ for Ta and Nb, respectively; while for 3Y-TZP is 12 × 10^−6^ °C^−1^. Thus, when these composites cool down from the sintering temperature, the reinforcement contracts less than the matrix; the metal particles are subjected to compressive stress and residual tensile stresses are accumulated in the zirconia matrix when cooling from the sintering temperature. When the residual tensile stress is imposed on t-ZrO_2_ grains, phase transformation can occur easily. Therefore, the phase transformation is promoted by an applied stress and the transformation rate increases with its magnitude.

Table 1 shows the monoclinic volume fraction estimated on polished and fractured surfaces of monolithic zirconia and zirconia/metal composites. The results show that in the monolithic ZrO_2_ only about 2 vol.% of the tetragonal zirconia transformed to the monoclinic phase during the failure. Moreover, the analysis of XRD data proved that the fraction of zirconia transformation considerably increases for zirconia/metal composites. The enhanced transformability can be related to the alloying effect on the tetragonality, i.e., the cell parameter ratio *c/a*, of stabilized t-ZrO_2_ as well^39^,^40^. The modest monolithic zirconia toughness value can be explained by the low transformation of zirconia after fracture (Table 1).

**Figure 5:**
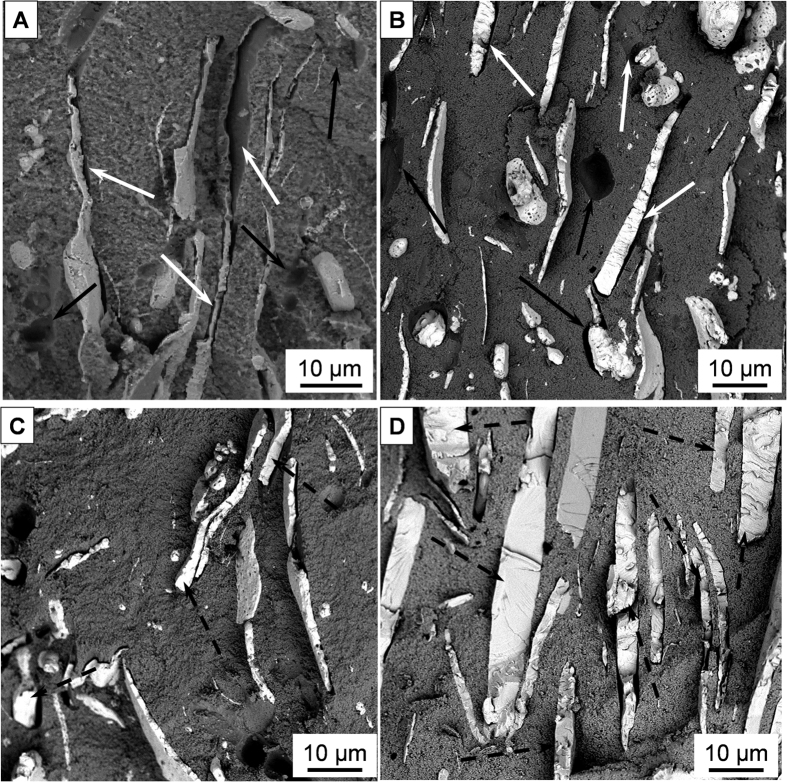
Fracture surfaces of SPSed ZrO_2_/Nb (A, C) and ZrO_2_/Ta (B, D) composites after bending and fatigue test, respectively. Black arrows show marks of rounded grains debonded from the brittle zirconia matrix. White arrows show decohesion between the matrix and the metallic particles. Dashed black arrows show the cleavage of metallic particles.

Analyses of the fracture surfaces of both ceramic/metal composites after bending tests indicate two distinct failure modes of the metal particles: i) the tantalum particles fracture surface bears little resemblance to typical ductile necking associated with void coalescence, such as those shown for niobium flakes (Fig. 5A–C), and ii) nearly planar facets form between ridges and valleys, reminiscent of brittle cleavage or intergranular decohesion have been observed mainly in tantalum flakes (Fig. 5B–D), in accordance with previous studies^42^.

**Figure 6:**
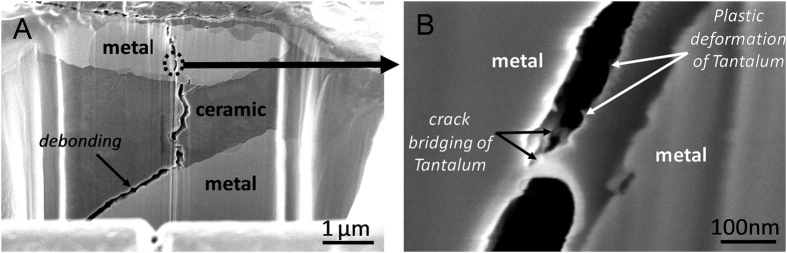
FIB-SEM image of crack propagation in the zirconia-tantalum composite. Arrows indicate interface debonding between the metal particles and the ceramic (**A**), plastic deformation (**B**) and crack bridging of ligament (**B**).

In order to investigate microstructural evolution of ZrO_2_/Ta composites with indentation crack propagation, FIB technique was used. Figure 6 shows the FIB-SEM image of crack-microstructure details along the crack path. The presence of micron-sized metal particles in the ceramic matrix leads to void nucleation, growth, and coalescence, either by particle fracture or by particle/matrix interface debonding. These observations also confirm the presence of plastic deformation and crack bridging of tantalum particles.

The lower slopes of the ceramic/metal composites in Fig. 3 indicate that these materials exhibit greater indentation strength and flaw tolerance than the monolithic ceramic. The explanation for this difference in slopes is that cermets exhibit better crack growth resistance (R-curve) behaviour in which the toughness increases with increasing crack length. Brittle materials showing no rising R-curve behaviour, present a slope of 1/3 (dashed line in Fig. 3) and materials with slopes lower than 1/3 are expected to present R-curve behaviour. For a given indentation flaw, ZrO_2_/Ta and ZrO_2_/Nb composites always showed higher values of fracture strength, which means that the damage tolerances of these composites are higher than for monolithic zirconia ceramic. Although un-indented ZrO_2_ ceramic exhibits the highest value of the fracture strength, the failure strength of the indented ceramic/metal composites is higher than in the monolithic zirconia.

The first conclusion that can be drawn from the obtained dependencies of stress amplitude *σ*_max_ vs cycles to failure *N* (Fig. 4) is that ZrO_2_/Nb composite exhibits fatigue behaviour in contrast to ZrO_2_/Ta composite and monolithic zirconia. This can be deduced from the fact that in the *S*–*N* plots the maximum stress (σ_max_) for ZrO_2_/Nb decreases with the cycles to failure *N*, while in case of ZrO_2_/Ta composite it slightly changes; and it is practically constant for ZrO_2_. Furthermore, the slope of the discussed dependency log *σ*_max_ vs log *N* is similar for the zirconia when compared to the ZrO_2_/Ta composites. Therefore, the sensitivity of ZrO_2_/Nb to cyclic stresses is higher than for the monolithic zirconia and for the ZrO_2_/Ta composites. Higher slope in Fig. 4 indicates higher accumulation of damage during the cyclic loading^43^. It can be concluded that SPS zirconia is too brittle, with no presence of any transformation toughening mechanism. This may be attributed to the nanoscale nature of the zirconia grains, below the critical transformation size. Thus, for all practical purposes, SPS zirconia does not show any evidence of cyclic fatigue behaviour. Cyclic fatigue effects in ceramic/metal composites are attributed to cyclic degradation of the bridging zone leading to a reduction of its shielding capacity. A “cyclic cleavage” mechanism has been proposed to explain this degradation in the case of bcc metals^44^,^45^. The increased strain rate associated with high frequency cyclic loading (20 Hz) promotes rapid fatigue-crack growth in the metal phase by “cyclic cleavage”, rather than by the ductile mechanisms seen in flexural strength tests (Fig. 5). Therefore, the early fatigue failure of metal particles limits the effectiveness of ductile-phase toughening under cyclic loading^46^. Crack trapping and renucleation of the fatigue crack in the ductile phase associated with blunting (via interfacial decohesion for metal particles), and coplanar bridging from multiple and discontinuous crack fronts are expected to provide the main contributions to the fatigue crack growth resistance^31^,^47^.

**Figure 7:**
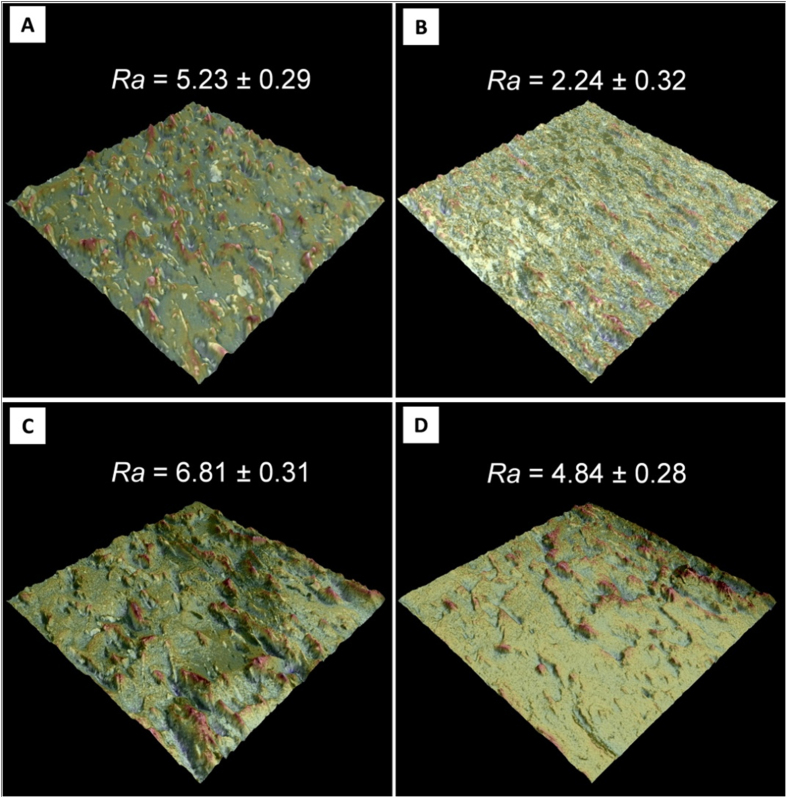
Composites’ fracture surface 3D images and corresponding roughness values (Ra, μm) after biaxial bending (A – ZrO_2_/Ta and C – ZrO_2_/Nb) and fatigue (B – ZrO_2_/Ta and D – ZrO_2_/Nb) tests.

**Figure 8:**
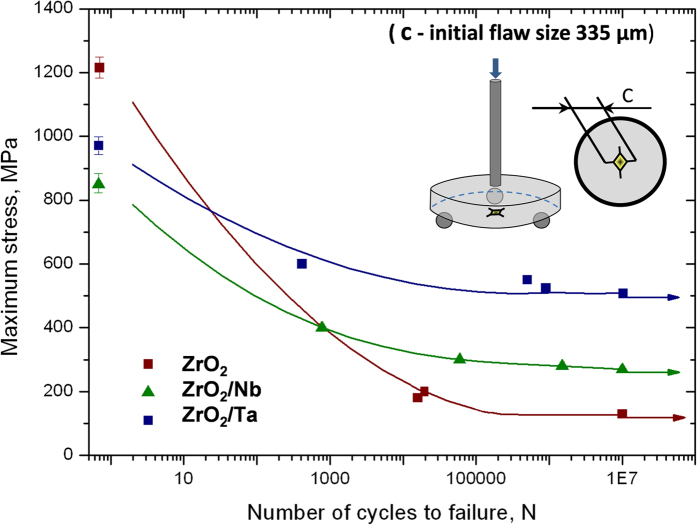
*S–N* plot of the cycles to failure (*N*) of indented ZrO_2_, ZrO_2_/Ta and ZrO_2_/Nb composites. Ultimate flexural strength of un-indented specimens is plotted as well.

Niobium has similar physical and chemical properties to those of the element tantalum but, it shows a lower elastic modulus than tantalum (105 GPa vs 186 GPa) and yield strength (240 MPa vs 380 MPa)^48^. Our simulations have shown similar interface strength values for the different ceramic/metal composites constituted by both Ta and Nb metals. Hence, the most significant mechanism to explain the differences in the fatigue curves for both composites must come from the relative weight of the elastic and plastic regime of the bulk metals. Therefore, niobium metallic particles show higher level of deformation under bending stress than tantalum metallic particles and, consequently, higher fracture surface roughness. Under a dynamic loading regime, tantalum failed by an elastic process which progressed by nucleation of scattered nanoscale voids, followed by void clusters growth and linking up with rather sharp cracks between voids showing little inter-void deformation, in contrast to the niobium behaviour where extensive void necking was observed. 3D roughness images corresponding to the composites fracture surfaces after biaxial bending and fatigue tests along with the corresponding roughness values (*Ra*), are presented in Fig. 7. After mechanical testing, the roughness values of the fracture surface of the ZrO_2_/Ta composites (Fig. 7A and B) are lower than the one corresponding to ZrO_2_/Nb material (Fig. 7C and D). Ra values are ≈25% and ≈55% lower in the case of fast and subcritical crack growth, respectively. This fact is a clear quantitative indication of the higher contribution of Nb ductility than Ta to the crack growth resistance during fracture. Also, niobium metallic particles show much higher level of plastic deformation under fatigue load than tantalum metallic particles. It is assumed that a steady state is reached resulting from the equilibrium between the shielding accumulation due to crack growth and the cyclic-induced degradation. In the case of ZrO_2_/Ta composite, the small gap between matrix and metal particle elastic modulus must play an important role. The crack tip energy was not used to plastically deform the metal particles as in the case of ZrO_2_/Nb composite, where the elastic modulus of Nb is nearly two times lower than the ceramic matrix. Then, the crack propagation resistance in this particular case (ZrO_2_/Ta) can be attributed to crack-particle interaction intrinsic mechanisms, including crack re-nucleation (in the matrix and/or particle), crack branching, crack blunting, limited interface debonding and discontinuous (out-of-plane) crack deflection. On the other hand, niobium and tantalum metallic particles showed lower level of plastic deformation under fatigue load than under bending stress and hence, lower roughness (Ra value ≈30% and ≈60% lower in the case of ZrO_2_/Nb and ZrO_2_/Ta composite, respectively). Therefore, crack particle interactions under cyclic loading are different than those observed under monotonic loading. Since fatigue crack growth occurs at stress intensities lower than critical crack growth, the toughening contribution from metals deformation would naturally be smaller than under monotonic loads. But the absence of significant plastic deformation of Ta particles in the ZrO_2_/Ta composite under cyclic loading is unexpected as metallic materials are known to be susceptible to fatigue. Thus, the presence of Ta reinforcements, while providing a potent influence on toughening zirconia ceramics under monotonic loading, is also effective in improving fatigue crack growth resistance. In order to predict fatigue life of indented materials with equivalent initial flaw size, indentation load of 98 N and 294 N were chosen for monolithic ceramic and for ceramic/metal composites, respectively. The initial flaw size was about 335 μm for all studied compositions. The indentation fatigue life for disc specimens are presented in semi logarithmic form as peak stress versus cycles to failure (Fig. 8). The indented specimens were tested under the same cyclic conditions as the un*-*indented ones.

It has been shown that fatigue life of well-polished ceramic/metal composites without any artificial produced flaw is reduced when compared with monolithic zirconia (Fig. 4). However results from Fig. 8 indicate that the situation changes dramatically for the indentation fatigue. In this particular case, it was found that the fatigue limit for ZrO_2_/Ta, ZrO_2_/Nb and ZrO_2_ is 500 ± 25 MPa, 270 ± 10 MPa and 130 ± 40 MPa, respectively.

In Table 2, the fatigue performance of all materials tested in the study is compared by the fatigue ratio (the ratio of the fatigue limit to the static strength of a material). For the monolithic ceramic, the ratio shows a significant decrease between un-indented and indented specimens. Therefore, the fatigue ratio of zirconia is extremely sensitive to surface condition and the presence of defects that act as stress raisers and, consequently, the chances of failure of monolithic ceramic is higher than in ceramic/metal composites. On the other hand, it has long been known that the fatigue ratio, is consistently higher for Ta (0.86) than for Nb (0.77)^49^. The ability to continue well-dispersed dislocation mobility, even after prolonged fatigue cycling, may be the most important characteristic of the Ta for fatigue resistance. A second reason for their superiority is the larger stress needed to move dislocations^36^. Hence, the yield stress of Ta will always be correspondingly higher which, together with the lower rate of work hardening in these metals, will place the fatigue limit closer to the strength.

**Figure 9:**
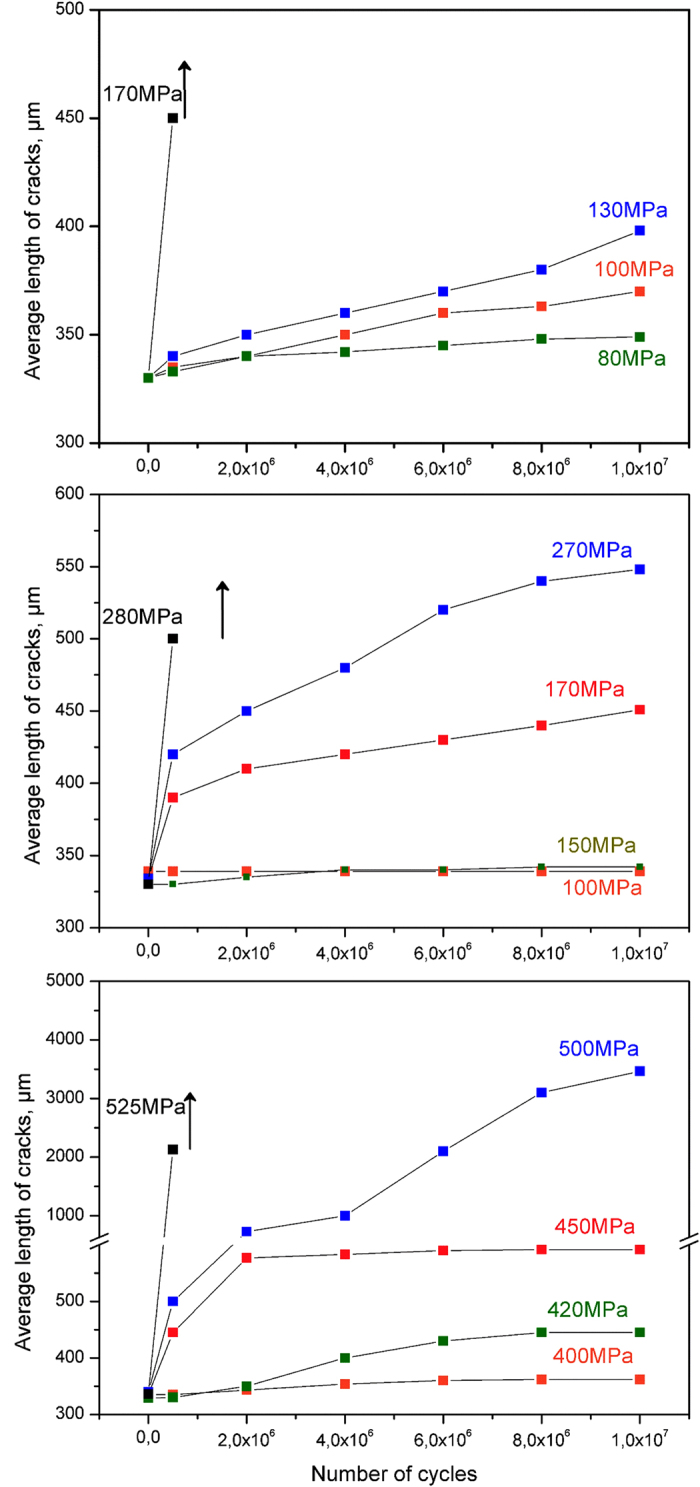
Number of cycles and crack length as a function of the applied load for ZrO_2_ (top), ZrO_2_/Nb (middle), and ZrO_2_/Ta (bottom) composites.

The strength and reliability of monolithic zirconia are significantly reduced when compared with composites. Meanwhile, less sensitivity of ceramic/metal composites to defects under cyclic loading, especially ZrO_2_/Ta materials, allows to visualize the growth of defects before reaching critical size and prevent sudden fracture. In order to observe crack propagation, the experiment was stopped at various stages of cycling tests. At each stop the test specimen was dismounted from the jig for SEM analysis. The combination of this information with the test load is showed in graphs of crack lengths as a function of the number of cycles (Fig. 9). In this figure, vertical black arrows correspond to the number of cycles of stress to cause complete fracture of the specimen. It has been found that zirconia ceramic with artificially induced flaws shows fatigue crack growth initiation when the applied load is only ~7% (80 MPa) of the flexural strength value of un-indented specimens. In other words, any flaw present in monolithic zirconia under very low subcritical loading can lead to catastrophic failure. In the case of ZrO_2_/Nb cermets, for load levels of ~17% (150 MPa) of flexural strength value of un-indented specimens cracks start to growth. For load levels as high as ~41% (400 MPa) of flexural strength value of un-indented specimens, the ZrO_2_/Ta composites show subcritical crack growth resistance and the cracks do not propagate. Moreover, fatigue crack length propagation for maximum stress under which the specimens never fracture (fatigue limit) is ~550 μm and ~398 μm for ZrO_2_/Nb and ZrO_2_, respectively. However, ZrO_2_/Ta composites show infinite fatigue life even in a severely damaged state (crack length of ~3500 μm) and therefore excellent flaw tolerance fatigue behaviour.

It was established that in contrast to fracture toughness, fatigue crack growth values do not exhibit a monotonic correlation with microstructural aspects. Zirconia/metal cermets reinforced with more ductile metal (Nb), were found to be more fatigue sensitive in terms of crack growth resistance. Moreover, these materials are more fatigue susceptible with artificial induced flaws (indentation) under cyclic loading. The experimental fact that failure under cyclic loading of zirconia/metal cermets is controlled by subcritical growth of preexisting flaws shows that a damage tolerance analysis for structural design involving these materials is difficult to apply in practice. In the case of ZrO_2_/Ta composites, there is a way to avoid the conflicts between mutually exclusive properties of toughness and fatigue resistance, through the presence of multiple mechanisms acting at different length scales, decreasing locally stresses through limited plastic deformation to provide intrinsic toughness and further extrinsic mechanisms, such as elastic bridging of the tantalum particles, with about double elastic modulus and yield strength value than Nb particles.

The results obtained in this investigation suggest that ductile phases are associated with multiple and competing mechanisms operating under fatigue loading and being sensitive to elastic and plastic properties (yield stress and elastic modulus) of the reinforcement. These properties and the interlocking operative mechanisms play a very important and critical role in the unexpected fatigue resistance of these ZrO_2_/Ta composites, considering the fact that materials with the more ductile metal are more fatigue sensitive. Cracks propagate only when the crack tip energy reaches a threshold that corresponds to the bond failure energy. If the metal particle-matrix bonds are both, strong and weak or the interface cleaves easily, the crack tip surface is multiplied (for instance by a particle circumference) and propagation stops since the stress intensity falls instantaneously below the above-mentioned threshold. In the case of Ta particle embedded in a zirconia matrix this threshold is much higher than in the case of more ductile Nb particles.

To our knowledge, the ability of tantalum metal to dramatically improve the fatigue resistance and damage tolerance of a ceramic material has not been previously reported in the literature, and was discovered here only because the particular microstructural feature of this ceramic/metal composite. These unprecedented properties presented in this work could, in principle, stimulate multidisciplinary applied research on ceramic/tantalum composites, which are attractive for various fields such as thermoelectric power generation, functionally graded materials, biomaterials, strain-tolerant and thermal-shock-resistant multifunctional ceramics, static-charge dissipation devices, electric-discharge manufacturing, and many more, and that open the doors for massive and sustainable utilization of cyclic fatigue resistance structures.

## Methods

### Starting materials

The following commercially available powders have been used as raw materials: (1) Tetragonal zirconia polycrystals (3Y-TZP, 3 mol% Y_2_O_3_; TZ-3YE, Tosoh Corp.), with an average particle size of d_50_ = 0.26 ± 0.05 μm, a BET specific surface area of 16 ± 3 m^2^/g. (2) Tantalum (Alfa Aesar, 99.97% purity) with an average particle size d_50_ = 44 μm. (3) Niobium (Goodfellow, Huntingdon, U.K., 99.99% purity) with an average particle size of d_50_ = 35 μm.

Metallic powders were attrition-milled with zirconia balls in a teflon container for 4 h using isopropyl alcohol as liquid media. The ball-milled resulting powder consists of flake-like deformed metallic particles with a high aspect ratio and a mean particle size of 42 μm and 41 μm of tantalum and niobium, respectively.

### Powder processing

In order to fabricate the zirconia matrix reinforced with lamellar Ta and Nb particles, 3Y-TZP powder was wet mixed with 20 vol.% of the ball-milled Ta and Nb powders and subsequently compacted by SPS at 1400 °C and 80 MPa in vacuum. Details of the ceramic/metal slurry processing and sintering parameters were reported elsewhere^23^,^29^,^50^. The sintered specimens had diameters of 20 and 50 mm and a thickness of 2–4 mm. For comparison, zirconia powder also was sintered under the same sintering cycle.

### XRD characterization

Tetragonal to monoclinic transformation measurements of zirconia were carried out in a Bruker D8 diffractometer using CuKα radiation (λ = 1.5405981 Å) working at 40 kV and 30 mA in a step-scanning mode from 27° to 33° with a step size of 0.01° and a scan speed of 0.06°/min. The amount of m-ZrO_2_ was evaluated from the diffractograms according to Garvie and Nicholson method^51^. Its volume fraction was calculated as proposed by Toraya H. *et al*.^52^. The error of XRD measurement was 1%.

### Microstructural and mechanical characterization

The microstructure of the sintered specimens was studied by Scanning Electron Microscopy (SEM, Phenom G2, Eindhoven, The Netherlands), Focused Ion Beam Scanning Electron Microscope (FIB-SEM) AURIGA 60 CrossBeam Workstation (Carl Zeiss Microscopy, Jena, Germany), and by transmission electron microscopy (TEM-JEOL 4000-EX, Tokyo, Japan). The TEM samples were prepared by diamond cutting and mechanical polishing to a thickness of 100 μm, followed by dimpling to a thickness of 20 μm and ion milling at 6.0 KeV and 0.5 mA. In order to determine the content of tantalum and niobium oxide solid solution in zirconia, scanning electron microscopy (SEM; Nova NanoSEM 230, FEI, Hillsboro, OR, USA) with energy dispersive X-ray spectroscopy (EDX) was using for chemical microanalysis of sintered samples. One hundred random points were chosen to obtain a semi-quantitative anlysis for the tantala and niobia distribution in the ceramic matrix.

The density of the compacts was measured according to the Archimedes technique. Methods to measure Vickers hardness (Hv), fracture toughness (K_Ic_), flexural strength (σ_f_) and Young modulus (E), formulas and calculation procedures have been reported in previous publications^23^,^41^,^53^.

The damage tolerance of the specimens was measured by analyzing the strength data for specimens as a function of indentation load. Indentation cracks are simple to generate and because they are sharp and rather short, they are believed to behave like natural cracks. Indentation strength tests were performed using the discs produced with the same preparation technique such as in flexural testing. The controlled Vickers indentation flaws generated at loads between 9.8 N and 490 N were placed at the centers of the tensile faces of each specimen.

The mechanical tests were performed immediately after indentation to avoid any subcritical crack growth due to stress corrosion effects. Special effort was made to examine all specimens after testing using reflected light optical microscopy, to verify that the indentation contact site acted as the origin of failure.

Fatigue life of specimens was determined not only for the ceramic/metal composites, but also for the monolithic zirconia for comparison purpose. Specimens with the same dimensions as the biaxial flexural strength test were subjected to a fatigue test using a SHIMADZU electromagnetic testing machine (EMT-1kNV-30, Kyoto, Japan), operating under load control at 20 Hz in order to generate a stress number curve. A sinusoidal cyclic load was applied to the specimens. The stress ratio was equal to 0.1 and the fatigue cycle was limited at 10^7^ cycles.

Indentation fatigue life was analyzed following the same protocol that in the case of the flaw tolerance measurements. Indentation prints have been introduced in the tensile surfaces of all of the test discs. In order to obtain equivalent initial flaw size load of 196 N and 294 N was selected for ceramic and in ceramic/metal composites, respectively. The crack length was measured discontinuously by means of an SEM microscope (Phenom G2, Eindhoven, The Netherlands).

Roughness reconstruction and coloured height map of the composites fracture surface after biaxial and fatigue tests were carried out by using SEM (Phenom G2, Eindhoven, The Netherlands) with the 3D Roughness Reconstruction application. The field of view area for the height map calculation was 490 μm^2^ (×550). Three samples of each material were scanned to evaluate the average surface roughness (Ra) of the fracture surfaces at five different locations.

## Additional Information

**How to cite this article:** Smirnov, A. *et al*. Unprecedented simultaneous enhancement in damage tolerance and fatigue resistance of zirconia/Ta composites. *Sci. Rep.* **7**, 44922; doi: 10.1038/srep44922 (2017).

**Publisher's note:** Springer Nature remains neutral with regard to jurisdictional claims in published maps and institutional affiliations.

## Supplementary Material

Supplementary Information

## Figures and Tables

**Table 1:** Densities and mechanical properties of the all studied specimens as well as volume fractions of tetragonal “t” and monoclinic “m” zirconia in polished and fractured surfaces and the resulting transformabilities of tetragonal zirconia.

Specimen	Density [% *φ*_*th*_]	Elastic modulus [GPa]	Flexural strength *σ*_*f*_ [MPa]	Hardness *HV* [GPa]	Fracture toughness *K*_*Ic*_ [MPa·m^1/2^]	Volume fractions of t- and m-ZrO_2_ [vol%]				Transformability of t-ZrO_2_ *V*_*trans*_
						Polished		Fractured		
						t	m	t	m	
ZrO_2_	99	198 ± 5	1217 ± 10	13 ± 0.3	6 ± 0.3	99	1	97	3	2
ZrO_2_/Ta	98	194 ± 7	970 ± 18	9 ± 0.7	16 ± 0.9	94	6	77	23	17
ZrO_2_/Nb	98	179 ± 6	850 ± 20	10 ± 0.8	15 ± 1	98	2	79	21	19

**Table 2:** Fatigue ratio of all tested materials.

Composition	Flexural strength *σ*_*f*_, [MPa]	Fatigue limit [MPa]	Fatigue ratio
Specimens without artificial flaws			
ZrO_2_	1217 ± 10	1200 ± 15	0.98
ZrO_2_/Ta	970 ± 18	860 ± 30	0.88
ZrO_2_/Nb	850 ± 20	370 ± 30	0.43
Artificially*-*flawed samples			
ZrO_2_	312 ± 7	130 ± 40	0.41
ZrO_2_/Ta	710 ± 15	500 ± 25	0.70
ZrO_2_/Nb	642 ± 17	270 ± 10	0.42
