# Supplementary material for: Unprecedented simultaneous enhancement in damage tolerance and fatigue resistance of zirconia/Ta composites
Source: Sci Rep. 2017 Mar 21;7:44922. doi: 10.1038/srep44922 (PMC5359604; doi:10.1038/srep44922)
Supplement: Supplementary Information [file srep44922-s1.pdf]

Supplementary Information

Unprecedented simultaneous enhancement in damage tolerance and fatigue resistance of zirconia/Ta composites

A. Smirnov, J. I. Beltrán, T. Rodriguez-Suarez C. Pecharromán, M.C. Muñoz, J. S. Moya and J. F. Bartolomé

Computational methodology

Density functional theory calculations[1] were performed for the ZrO_2/Ta and ZrO_2/Nb interfaces using the *ab-initio* SIESTA code[2] within the generalized gradient approximation (GGA)[3]. The basis set for the Nb, Ta, Zr and O have been described previously [ref 16 in the main text]. The ZrO_2-metal interfaces have been modeled within the supercell approach with periodic boundary conditions. Symmetric ZrO_2 and metal slabs form the supercell, which therefore contains two identical interfaces. We have calculated the low index (100)/(100) and (111)/(100) orientations for both ZrO_2/Ta and ZrO_2/Nb and the (3-30)/(1-1-2), repetitively observed in the HRTEM images, for the ZrO_2/Ta system. The number of ZrO_2 and metal layers forming the supercell depends on the interface orientation and are 11/8, 9/5 and 4/6 for the (100)/(100), (111)/(100) and (3-30)/(1-1-2) supercells, respectively. The selection of the relative in-plane vector alignments of the ZrO_2 and metal slabs was performed considering the smallest commensurate interface two-dimensional (2D) unit cells and the relative positions of the atoms were chosen in such a way that the number of metal-ceramic bonds is maximized. The equilibrium structures were obtained allowing relaxation of both, the unit cell and all the atomic positions, until the atomic forces are less than 0.05 eV/Å. The supercell Brillouin zones (BZ) were sampled with the Monkhorst-Pack grid and the number of *k*-points in the 2D BZs was always larger than 24. Further details on the calculations can be found in ref. [16] of the main text.

Interface structure and bonding

Figure S1 shows the equilibrium atomic structure of three representative ZrO_2/Ta interfaces. As can be observed, the shortest bonds and one of the largest density of bonds at the interface, 0.157 bonds/$Å^2$, occur for the (100)/(100) orientation, while the (111)/(100) and (3-30)/(1-1-2) interfaces present larger bonds and smaller or similar bond densities 0.137 and 0.160 bonds/$Å^2$, respectively. Hence, the (100)/(100) seems to be stronger. In order to quantify the strength and stability of the investigated ZrO_2/Ta interfaces, we calculate the work of separation, *Wsep*, and the interface free energy, $\gamma_{ZrO2\text{-}Ta}$ [4,5]. The ideal *Wsep* is defined as the reversible work needed to separate the interface into two free surfaces, if the plastic and diffusional degrees of freedom are supposed to be suppressed. While the interface free energy, $\gamma_{ZrO2\text{-}Ta}$, gives the excess free energy of an interface compared to the corresponding bulk materials. It allows one to know which interfaces are more stable in a thermodynamic sense. Both quantities can be obtained from the calculated *ab-initio* total energies corresponding to the ZrO_2 and Ta bulk systems as well as those from the free surfaces and from the surfaces in contact forming the interface. The *Wsep* is independent of the environmental condition, but the interface free energy for non-stoichiometric interfaces, as those formed with the polar (100) ZrO_2 orientation, depends on the surrounding atmosphere. Since changes in the composition of the non-stoichiometric polar surfaces take place in order to adjust the concentration of Zr and oxygen to values more favourable for the free surfaces. The calculated *Wsep* represents a lower bound for the actual fracture energy involved in any experiment, since due to dissipative processes, the measured energy will always exceed the ideal value obtained from *ab-initio* calculations. Moreover, the thermodynamic stability of any ZrO_2/metal interface is related to the inverse of the interface free energy -the lower the interface energy the higher the stability-. Therefore, by comparing the *Wsep* and the $\gamma_{ZrO2\text{-}Ta}$ of different surfaces their relative bonding strength and stability can be determined -for a detailed discussion of the *Wsep*, the stability of the interface and their correlation with the interface strength[5]. Table S1 shows the *Wsep* for several interfaces of the ZrO_2/Ta and ZrO_2/Nb composites. Both systems present similar works of

separation for analogous crystallographic directions, although the values are slightly larger for the Ta-based interfaces. The *Wsep* is very different depending on the polarity of the ZrO_2[4]. The polar ZrO_2(100) surface has a large number of dangling bonds per unit area and consequently interfaces formed with this orientation exhibit very high *Wsep* due to the avidity of ZrO_2 surface oxygen to saturate and thus to form strong metal-oxygen bonds. The *Wsep* correlates also with the number of short interface bonds and bond density per interface area, as it is indicated in Figure S1 for the ZrO_2/Ta composite. Opposite, the (111)/(100) and (3-30)/(1-1-2) with larger bonds and smaller or similar densities exhibit smaller *Wsep.* In addition, Figure S2 shows that the weaker non-polar ZrO_2, interfaces are the more stable, while the stronger polar ones, i.e. (100)/(100) orientations, are less stable in all the energy range of oxygen chemical potential and hence in all the range of experimental conditions. Accordingly, the ZrO_2/metal composites should exhibit an average moderate interface strength.

1. Koch, W. & Holthausen M.C. *Chemist's guide to density functional theory*. (Wiley-VCH; 2001).

2. Soler, J.M. *et al.* The SIESTA method for ab initio order-N materials simulation. *J. Phys.: Condens. Matter.* **14**, 2745-2779 (2002).

3. Perdew, J.P., Burke, K. & Ernzerhof M. Generalized gradient approximation made simple. *Phys Rev Lett*. **77**, 3865 (1996).

4. Beltrán, J.I. and Muñoz, M.C. Ab-initio study of the decohesion properties in oxide/metal. *Phys. Rev. B* **78**, 245417 (2008).

5 Muñoz M.C., Gallego S., Beltrán J.I., Cerdá J., Adhesion at metal–ZrO_2 interfaces, Surf. Scien. Rep. **61**, 303 (2006).

Table 1. Work of separation (*Wsep*) in J/m^2 and interface metal-oxygen shortest bond distances in Å for several ZrO_2/metal interfaces. The ZrO_2/Nb values correspond to those in Ref.16 of the main text [*].

**ZrO_2-M interface**	**(111)-Ta(100)**	**(100)-Ta(100)**	**(3-30)-Ta(1-1-2)**	**(111)-Nb(100)$_{[*]}$**	**(100)-Nb(100)$_{[*]}$**
$d_{M\text{-}O}$ (Å)	2.15	2.05	2.16	2.22	2.10
W_{SEP} (J/m^2)	2.23	10.18	2.22	1.84	9.02

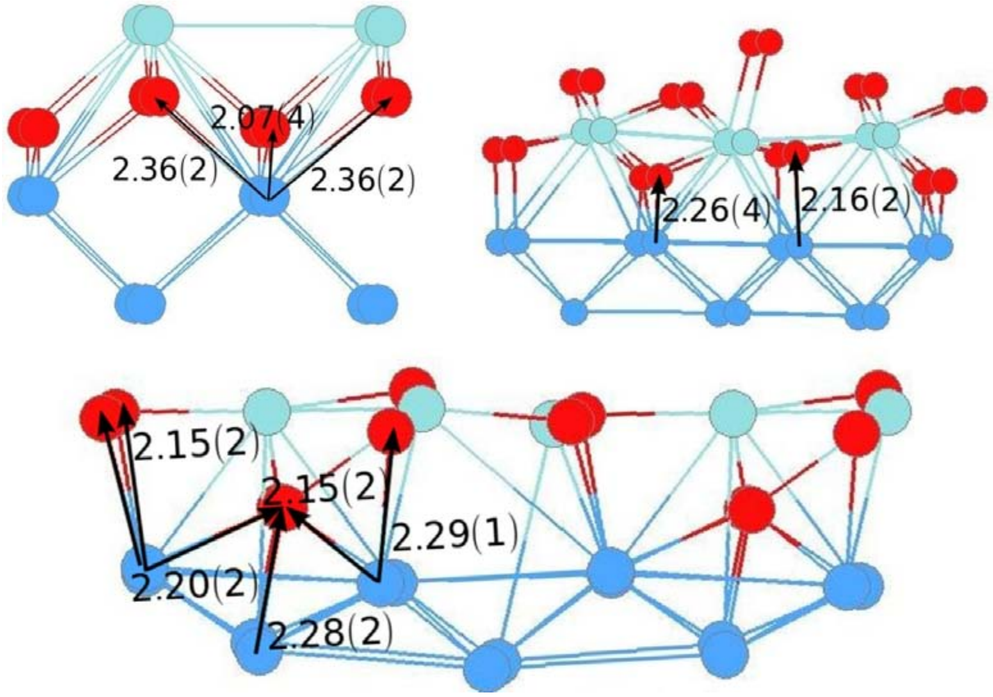

Figure S1. Schematic representation of the atomic structure of ZrO_2/Ta interfaces: a) (100)/(100), b) (111)/(100) and c) (3-30)/(1-1-2) orientations, respectively. The colour code corresponds, cyan to Zr, red to oxygen and blue to metal atoms (Ta or Nb). The arrows show the shortest metal-oxygen bond distances and in brackets the number of similar bonds occurring in the two-dimensional (2D) unit cell is given. The areas of 2D unit cells of the different interfaces are a) 51.00, b) 43.71 and c) 55.79 Å$^{2.}$

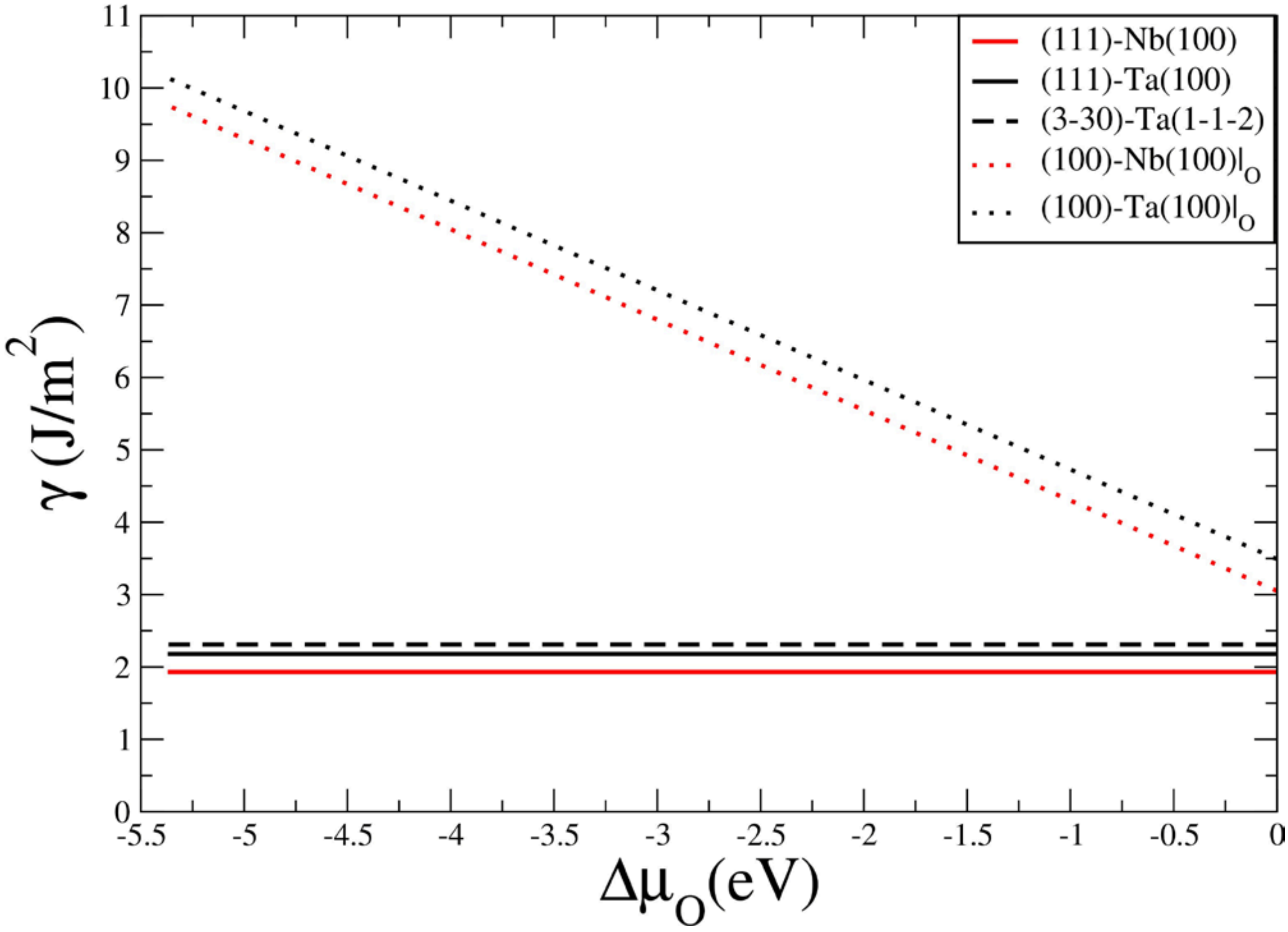

Figure S2. Interface free energy versus the oxygen chemical potential $\Delta\mu_0$. The interface free energy is obtained from the Gibbs free energy of the complete interface system and the bulk energies of the ZrO_2 and the metal. The latest are given by the number of the corresponding atoms in the interface unit cell and their chemical potential, see ref [5]. $\Delta\mu_0$ is the difference between the oxygen chemical potential in the ZrO_2 and in the gas phase. It ranges between zero and half the heat of formation of $ZrO_{2.}$ For a detailed discussion of the interface free energy and its relation to the stability of the interface see ref. [5].
